# Personalised 3D Printed Medicines: Which Techniques and Polymers Are More Successful?

**DOI:** 10.3390/bioengineering4040079

**Published:** 2017-09-22

**Authors:** Andrea Alice Konta, Marta García-Piña, Dolores R. Serrano

**Affiliations:** 1 Departamento de Farmacia y Tecnología Farmacéutica, Facultad de Farmacia, Universidad Complutense de Madrid, Plaza Ramóny Cajal s/n, 28040 Madrid, Spain; akonta@ucm.es (A.A.K.); martga08@ucm.es (M.G.-P.); 2 Instituto Universitario de Farmacia Industrial (IUFI), Facultad de Farmacia, Universidad Complutense de Madrid, Avenida Complutense, 28040 Madrid, Spain

**Keywords:** 3D printing, drug delivery, personalised medicine, polymers, FDM, SLA

## Abstract

The interindividual variability is an increasingly global problem when treating patients from different backgrounds with diverse customs, metabolism, and necessities. Dose adjustment is frequently based on empirical methods, and therefore, the chance of undesirable side effects to occur is high. Three-dimensional (3D) Printed medicines are revolutionsing the pharmaceutical market as potential tools to achieve personalised treatments adapted to the specific requirements of each patient, taking into account their age, weight, comorbidities, pharmacogenetic, and pharmacokinetic characteristics. Additive manufacturing or 3D printing consists of a wide range of techniques classified in many categories but only three of them are mostly used in the 3D printing of medicines: printing-based inkjet systems, nozzle-based deposition systems, and laser-based writing systems. There are several drawbacks when using each technique and also the type of polymers readily available do not always possess the optimal properties for every drug. The aim of this review is to give an overview about the current techniques employed in 3D printing medicines, highlighting their advantages, disadvantages, along with the polymer and drug requirements for a successful printing. The major application of these techniques will be also discussed.

## 1. Overview

The interindividual variability is an increasingly global problem when treating patients from different backgrounds with diverse customs, metabolism, and necessities. Currently, dose adjustment is frequently based on empirical methods, and therefore, the chance of undesirable side effects occurring is high. For example, in the United States, millions of side effects are expected annually, leading to over 100,000 deaths [1].

Over the years, such variability has been accepted as part of the therapeutic process, but nowadays, new technologies allow for the optimization of treatments according to population subgroups, based on pharmacogenetics and pharmacokinetic profiles [1].

The aforementioned science pretends to elude the traditional inclination of the Pharmaceutical Industry to manufacture drugs according to the most abundant and representative therapeutic response profile. Drug mass-production impedes developing medicines when taking into account all of the particularities of every individual. This fact is especially relevant on paediatric and geriatric populations, leading to inadequate treatment doses and a high probability of adverse effects. Dose adjustment according to pharmacogenetic and pharmacokinetic characteristics, weight, and age are key in order to achieve the desired therapeutic effect and to improve the efficacy/toxicity balance. Likewise, a modification of colors, flavors, even the form of the solid dosage forms, would considerably increase the adherence to treatment both in children and elder people [2].

But not only children and elderly suffer from the inadequacy of solid dosage forms adapted to their necessities; polimedicated patients, those who have prescribed more than five different types of medicines, are also at risk of therapeutic failure and the promotion of side effects and drug interactions due to poor patient compliance and treatment adherence. Simplification of drug therapy based on the intake of a single pill containing all of the drugs required for the patient would improve adherence to the prescribed treatment and, probably enhance the efficacy/toxicity balance [3].

There is evidence of the multiple therapeutic gaps that prevail with the conventional manufacturing of drugs, demanding a restructuration of the drug production with the purpose of promoting the personalisation of pharmacological treatment. From the technological point of view, this progress is getting place due to the continuous modification of drug delivery systems, from traditional to the nanomedicine, as well as targeted therapies that have managed to increase the specificity of pharmacological treatments, thus reducing their toxicity.

But the trend that is really revolutionizing the Pharmaceutical Technology field is the Rapid Prototyping (RP), which was developed with the aim of reducing the tedious periods of time and the enormous costs in drug development and manufacture. RP lies in the fabrication of physical models using computer-aided design (CAD) information in three dimensions. It includes very different technologies, but all of them fall under the denomination of “additive manufacture” (AM) or the “manufacture of solids of free form” (SFF), including one of the most avant-garde technologies: three-dimensional (3D) printing. This new method brings the Pharmaceutical Industry into a new context, giving the possibility for tailored drugs and personalised treatment [4].

The first 3D printing technique used in pharmaceutics was achieved by inkjet printing a binder solution onto a powder bed, binding therefore the particles together thanks to the semi-liquid binding solution. The process was repeated until the final desired structure was obtained. This first happened in the early 90’s at the MIT (Massachuset Institute Technology) invented by Sachs et al. Nowadays, inkjet printing has come a long way, as it is the method used to manufacture Spritam, the first 3D printed drug approved by the Food and Drug Administration (FDA) and released to the market in the summer of 2016 by Aprecia Pharmaceuticals [5]. In the previous decade, in 1989, Scott Crump, filed a patent on another 3D printing technology: fused deposition modeling (FDM), where extruded polymer filaments heated into a semi-liquid state were extruded through a heated nozzle and deposited onto a build platform layer by layer to harden [5,6]. Since then, many other 3D printing techniques have been developed.

In this review, it will be discussed which are the most suitable techniques for 3D printing of medicines attending to the physicochemical characteristics of the drugs as well as which polymers are better candidates, and which applications can derived from these techniques.

## 2. Current 3D Printing Techniques

### 2.1. Printing-Based Inkjet Systems

Printing-based inkjet systems encompass two types of techniques: continuous inkjet printing (CIJ) and drop-on-demand (DOD) printing (Table 1). Both of the techniques are based on Lord Rayleigh’s Theory of instability of 1878, which explains the rupture of a stream of liquid or jet in drops [6].

**Figure 1:**
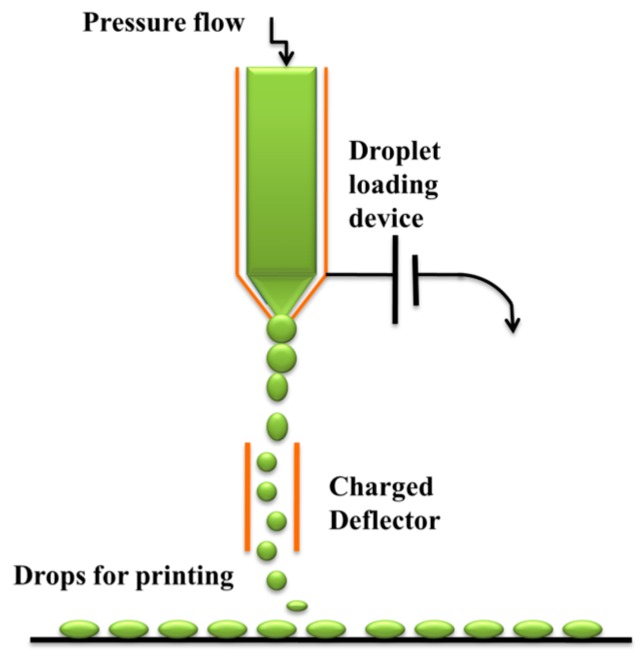
Continuous inkjet printing (CIJ).

In the first case, a high-pressure pump directs the liquid ink through an orifice between 50 and 80 μm in diameter, creating a continuous ink flow. A piezoelectric crystal causes the liquid to flow in order to break into drops at a specific speed and size and at regular intervals of time. An electrostatic field is created to control these parameters. Thus, the droplets are charged and separated by “droplets of guard” to minimize the electrostatic repulsion between them. The charged droplets are directed to the substrate due to the electrostatic field that has been created (Figure 1) [7]. 

In the continuous mode, the droplets are driven incessantly based on a counter-mechanism of injection on demand, as they are expelled when necessary. The DOD technique contains multiple heads (100–1000) and can use two types of translators, either a thermal head or a piezoelectric crystal. The thermal head is restricted only to volatile liquids, whereas the piezoelectric covers a wide range of liquids [4]. In addition, the thermal head reaches temperatures of up to 300 °C, which implies that the use of solvents of high vapor pressure could cause the degradation of bioactive compounds. This factor limits the use of thermal print heads for pharmaceutical applications [8]. The piezoelectric crystal changes rapidly, but this can generate a sudden variation of volume. Both of the heads are capable of producing droplets of between 10 and 50 μm, corresponding to a volume of between 1 to 70 pL [4,7]. The ability to operate at room temperature, with less volatile and more biocompatible liquids, makes piezoelectric printing technology more suitable for the development of drug delivery devices and medicines [8].

**Figure 2:**
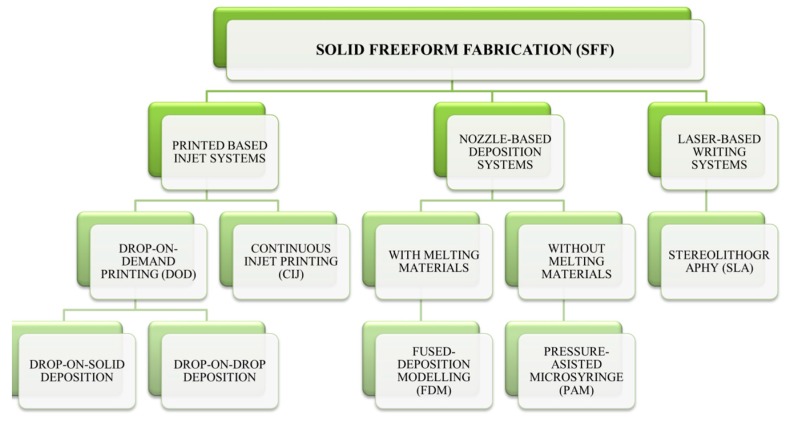
3D printing technologies for medicine manufacture. Modified from reference [4].

The DOD technique is divided into two subtypes: drop-on-drop deposition and drop-on solid deposition (Figure 2). Both of the systems have the advantage of using multiple materials and colors since they are able to print different pieces at the same time layer by layer and the printing time is reduced. In the drop-on-drop deposition system, the drop interposition produces the different layers of the RP. In contrast, the drop-on-solid deposition, known as “powder bed fusion” is based on the projection of drops directly on the solid material. It is optimal for a wide range of active ingredient substances (APIs), allowing the control of the drug release systems depending on the chemical properties of the binder used (Table 1). The latter technology works with two key elements for the process: the powder base, which simulates the “bed”, and, the binder that works like the “ink”. A technique derived from the latter on is the selective laser sintering (SLS), which instead of using a liquid ink, employs a laser to bind the powder particles together [4,8,9]. 

In both of the techniques, it is essential to use a heat post-treatment of the three-dimensional manufactured product in order to eliminate solvents used during the process being of advantage to avoid impurities and solvent residuals within the printed medicines [4]. However, the big drawback of the process lies on the fact that the three dimensional structures are very fragile and irregular. The fragility is justified by the high porosity of the conformations, as well as the extremely large diameter of the particles and the space between each of the lines, which explains the unevenness of the surfaces in the product created [6].

### 2.2. Nozzle-Based Deposition Systems

New technologies have been developed to overcome the limitations of the previous technology. Nozzle-based deposition systems consist on the mixing of drugs and polymers and other solid elements prior to 3D printing. The mixture is passed through a nozzle that definitely originates, layer by layer, the three-dimensional product. There are two types of printings according to the type of material used: FDM (Fused Deposition Modelling), which uses melted components, and, PAM (Pressure-Assisted Microsyringes), which does not require the use of melted materials (Table 1) [4].

#### 2.2.1. Fused Deposition Modelling (FDM)

FDM printing is based on the use of thermoplastic polymers such as polylactic acid (PLA), acrylonitrile butadiene styrene (ABS), or polyvinyl alcohol (PVA) [6]. However, ABS is not suitable for three-dimensional printed medicines, because it is not a biodegradable polymer (Table 2) [13].

**Figure 3:**
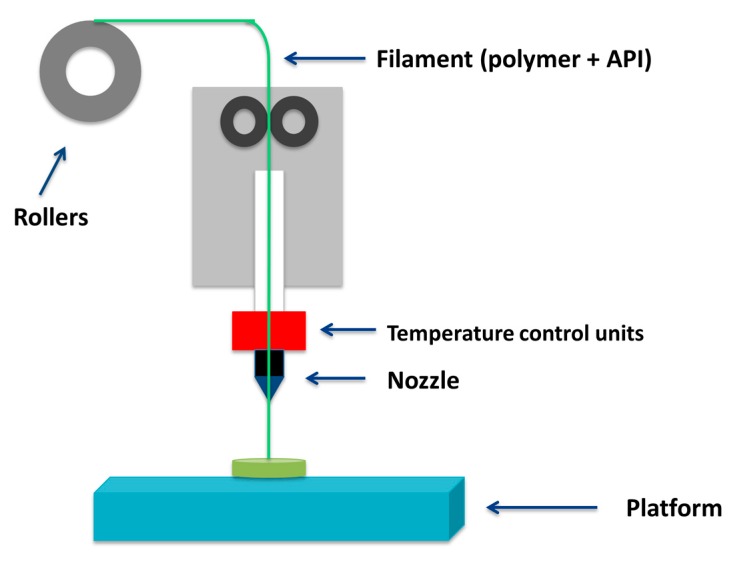
Fused deposition modelling (FDM) Printing system.

Melted materials (API and polymer mixtures) are stored in rolls arranged in such a way that they pass through an extruder nozzle as the process progresses. The nozzle is above the melting temperature of the material, and as a consequence the polymer-API mixture melts and deposits, layer by layer, in the form of fine filaments that are immediately solidified. This is why it is also called Fused Filament Fabrication (Figure 3). To facilitate the processing, materials must have adequate rheological properties. These properties are influenced by the nozzle diameter, the pressure drop, the feed rate, and others factors related to the thermal properties of the feed material, such as thermal conductivity, density, or glass transition temperature (Tg) [21]. 

An advantage of FDM over powder-bed printing is its higher resolution, which allows it to produce more complex scaffolds, and to achieve a better dosing accuracy. Besides this, FDM also offers good mechanical strength and the option to obtain different release profiles of the printed dosage forms by modifying the infill percentage, the 3D model design, or the surface area of the formulation. Some drawbacks are the limited thermoplastic materials options with good melt viscosity properties for extrusion, and the inability to use some APIs due to the high temperatures of the process [5].

Overall, it is the most commonly used 3D printing technique under research because of its ability to create complex drugs with difficult geometries, but the quality and speed of manufacture need to be improved to make it successful in clinical practice. 

#### 2.2.2. PAM Technology

This technology is based on the use of a syringe extruder that deposits a viscous and semi-liquid material, by means of a pressurized air piston, layer by layer, according to the designed geometry. Viscosity, viscoelasticity, and the apparent elastic limit are the keystone parameters that determine the reproducibility of this technique. It has advantages over other processes, as it has the possibility to work with a continuous flow and at room temperature. However, it also presents disadvantages, like the use of solvents, which are often toxic to health and cause a loss of stability in certain APIs. Its most useful applications are related to tissue printing substitutes or scaffolds of soft tissues, as well as the manufacture of complex drug delivery systems [4,22].

### 2.3. Laser-Based Writing System

**Figure 4:**
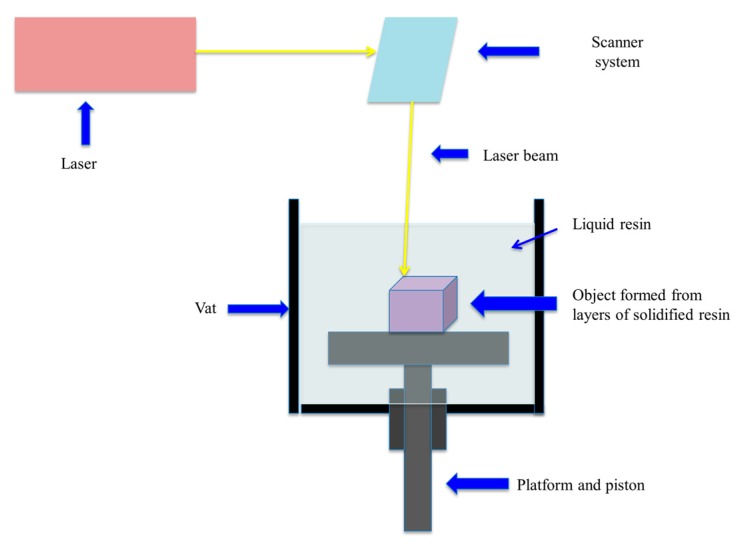
Stereolithography (SLA) printer.

It is the basis of the first device invented for the manufacture of three-dimensional products. It was designed by Charles Hull in 1980 and it was called “Stereolithography” (SLA) [23]. SLA printers are composed of an ultraviolet light beam, in the form of a laser, which transfers the energy into a liquid photopolymerizable resin [21]. In fact, it is based on the principle of photopolymerization, in which free radicals are released after the interaction between the photoinitiator and UV light [4]. The ultraviolet light beam is aided by baffles, axes *x* and *y*, to traverse the surface of the liquid resin, in order to accurately represent the 3D model, previously designed (Figure 4) [24]. When a layer solidifies, the lifting platform descends its position to the height of a new layer of liquid resin, again beginning the procedure, until the manufacture of the 3D product is finished in a layer-by-layer way.

An important parameter in SLA is the thickness of the cured layers, which can change depending on the energy of the UV light to which the resin is exposed to. Also, the choice of the resin is essential too, as it has to be a liquid biomaterial ready to solidify quickly upon illumination with the laser light and has to be FDA-approved for human use [4,5]. SLA stands out as a versatile option, as the drug and the photopolymer can be mixed prior to printing, becoming trapped in the solidified matrix. Other advantages are its high resolution over the other techniques and that heating is minimized during printing, which allows for the use of thermolabile drugs unlike FDM. The main disadvantages are that there are not too many options of photopolymers to choose. These materials are not currently considered safe in human use and the drug loading is low. Some polymers for this technique developed during the last few years are Poly(ethylene glycol) diacrylate (PEGDA), poly(2-hydroxyethyl methacrylate) (pHEMA), poly(ethylene glycol), dimethacrylate (PEGDMA), and poly(propylene fumarate)/diethyl fumarate (PPF/DEF) [5].

Overall, SLA is the type of 3D printing most widely used in medical bioengineering due to the characteristics of the three-dimensional products that are obtained [4,25,26]. 

## 3. Polymers Used in 3D Printing for Medical Purpouses

### 3.1. Polyvinyl Alcohol (PVA)

**Figure 5:**
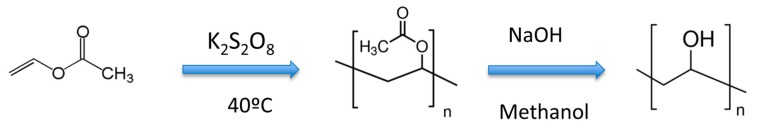
Polyvinyl alcohol (PVA) synthesis.

Polyvinyl alcohol (PVA) is a thermoplastic synthetic polymer with good solubility in water, low solubility in ethanol and insoluble in many organic solvents. It is unsavoury, scentless and has good mechanical properties. It is produced by either partial or full hydrolysis of polyvinyl acetate by removal of the acetate groups (Figure 5) [27,28]. The level of hydroxilation has an influence over the mechanical, chemical and physical properties of the polymer. Depending on the hydrolysis degree of the acetate groups, the melting point (Tm) of PVA ranges from 180 °C (partially hydrolyzed) to 220 °C (fully hydrolized). Hydrolization degree sets the viscosity scale of the polymer ranging from 3.4 to 52 mPa·s for partially hydrolized PVA to 4 to 60 mPa·s for fully hydrolized PVA [4,27]. The lower the degree of hydrolysis and polymerization of PVA, the higher its solubility in water and the easier its crystallization [27]. Also, the molecular weight of the polymer is higher when the degree of hydrolization is lower [28]. PVA has a glass transition temperature (Tg) of 85 °C while its degradation temperature ranges from 350–450 °C.

Because of its water solubility, PVA needs to be crosslinked to form hydrogels, as the crosslinks give the stability to the structure of the hidrogel after swelling in the presence of water or biological fluids. The degree of crosslinking determines the difussional and biological properties of the polymer. PVA has poor gatrointestinal absorption being classified as non-toxic polymer along with the fact that it has a high oral LD_50_ (from 15 to 20 g/kg). Its good biodegradability and its lack of adverse effects make it suitable for biomedical pharmaceutical applications (Table 3) [27].

One of the applications is in 3D printing. PVA has been used to produce multilayers of the polymer for additive manufacturing by the inkjet printing method (XYPrint100Z Hybrid printer with Konica Minolta KM512 print head). The formulated ink consisted of aqueos solutions of PVA with humectant (glycerine or monopropylene glycol) to avoid nozzle blockage and pigment (duasyn acid violet). Other inks were prepared with a combination of high and low molecular weight PVA. The molecular weight affects the inkjet printability due to the ink viscosity. Inks prepared from high molecular weight PVA did not aquire any color and mantained a good stability over six months. On the contrary, inks form lower molecular weight PVA formed gels with a milk-like appearence after six months, demonstrating that the requirements were not satisfied for inket printing. Noteworthy is that all of the inks showed a combination of pseudoplastics and thixotrypic behaviour at low shear rates and Newtonian behaviour at high shear rates [29].

Besides inkjet printing, PVA has also been successfully used in FDM. When using this technique, the parameters that need to be carefully controlled are the infill density (which ranges from 0% for hollow structures until 100% for completely solid cores), the speed of the extruder, the height of the layers, and the temperature of nozzles and building plate. The standard speed for FDM is 90 mm/s and the range of thickness travels from 100 to 400 μm. In some cases, PVA filaments are loaded by impregnation and incubation in a saturated solvent solution containing a dissolved API. After this incubation period, filaments are dried and then used for printing [4].

Several publications shave shown that PVA filaments can load until 10% drug content. For example, Goyanes et al. loaded PVA filaments via hot melt extrusion with paracetamol and caffeine. A FDM printer with the following settings: 200 °C extrusion temperature, 100% infill percentage, and 90 mm/s extruding speed was used to prepare oslid dosage forms containing a drug loading ranging from 4 to 10%. Drug release was decreased in those filaments with a lower API loading [30].

### 3.2. Poly(Lactic Acid) (PLA)

Poly(lactic acid) is a biodegradable polymer recognized as safe (GRAS) by the United States Food and Drug Administration (FDA) and suitable for different medical applications, such as: tissue engineering, regenerative medicine, drug delivery systems, wound management, stent applications, orthopedic, and fixation devices. The main production techniques for this polymer are based on directand ring opening polymerization [31]. The properties vary depending on the ratio of isomers, the processing temperature, the molecular weight and the cristallinity of PLA. The cristallinity refers to the amount of crystalline and amorphous regions of the polymer and influences certain characteristics such as hardness, melting point, stiffness, or tensile strength. PLA homopolymer has a melting point of 150–175 °C and a Tg of 55 °C [6,31]. At 200 °C, PLA has a reported melt viscosity of 1000 Pa·s, while it can reach 5100 Pa·s if shear stress and elevated temperatures are applied.

PLA and its derivates have good solubility in dioxane, acetonitrile, chloroform, methylene chloride, 1,1,2-trichloroethane, and dichloroacetic acid. All of them have low solubility in cold ethyl benzene, toluene, acetone, and tetrahydrofuran, but solubilty is increased when the solvents are heated and reach boiling temperatures. Poor solubility has been reported in water, alcohols (e.g., methanol, ethanol), propylene glycol, and unsubtituted hydrocarbons (e.g., hexane and heptane) [31]. 

**Figure 6:**
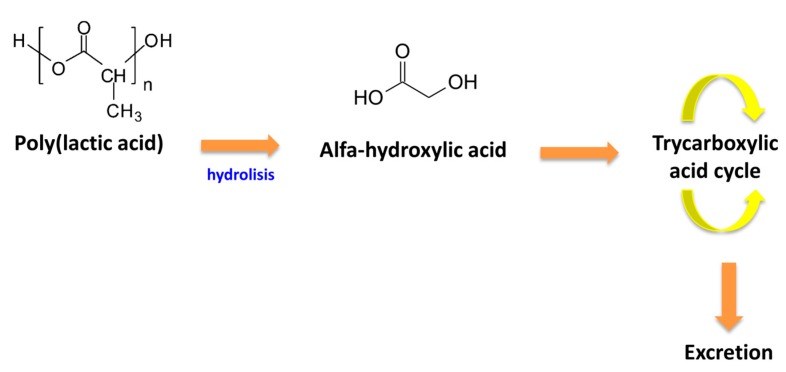
Polylactic acid (PLA) degradation in the human body.

One key point of PLA for medical applications is its capability of not producing toxicity or carcinogenic effects on the human organism, and not metabolisimg in toxic degradation products. When introduced in the human body, PLA hydrolizes to alfa-hydroxy acid. Following this step, the product enters into the tricarboxylic acid cycle and it is then excreted (Figure 6) [31]. The degradation rate of the polymer depends mainly on the following factors: crystallinity, molecular weight, and stereochemistry. Besides that, water diffusion into the polymer, distribution, and morphology determine the degradation rate, even though they have less impact on it. Overall, PLA has a slow degradation time, which leads to long in vivo life-time [31,32]. The period in which the polymer mass reaches zero in a saline enviroment at 37 °C can be up to 3–5 years and the interval in which the tensile strenght of PLA becomes 50% in a saline enviroment at 37 °C is between 6 to 12 months [33]. Copolymerization with PLLA modifies the degradation rate of PLA. PLA blends with PLLA increasing the degradation time, as the D-lactic acid is not easily degraded by the enzymes in the human body [34]. In contrast, copolymerization with polyglycolide increases the amount of amorphous domains in the polymer, thus being this copolymer easier to degrade (degradation time between 5–6 months) [32].

Regarding thermal degradation, it can take place by hydrolisis, lactide reformation, oxidative scission of the main chains, and other processes. The degradation is a simple process, taking place in one step, losing 5% mass polymer at 325 °C, and not remaining any residue at 500 °C [32]. 

As a cause of its hydrophobicity, PLA displays a low cell affinity, which can lead to an inflammatory response from the host when it has a direct contact with biological fluids. Another characteristic is that it is more brittle than other polymers [31].

Due to its properties (Table 4), PLA has been successfully used in medical devices using different 3D printing techniques such as FDM or laser based techniques [22].

### 3.3. Poly(Caprolactone) (PCL)

Poly(caprolactone) is a semi-crystalline hydrophobic polymer whose crystallinity tends to increase as its molecular weight decreases. Its melting point ranges between 59–64 °C, and it has a Tg of −60 °C [34,35]. PCL has good solubility in chloroform, dichloromethane, carbon tetrachloride, benzene, toluene, cyclohexanone, and 2-nitropropane at room temperature. In contrast, it has low solubility in acetone, 2-butanone, ethyl acetate, dimethylformamide, and acetonitrile and it is insoluble in alcohol, petroleum ether, and diethyl ether (Table 5) [36]. PCL blends well with many different polymers as poly(vinyl chloride), poly(styrene-acrylonitrile), poly(acrylonitrile butadiene styrene), poly(bisphenol-A), and it is mechanically compatible with others such as (polyethylene, polypropylene, natural rubber, poly-(vinyl acetate), and poly(ethylene-propylene) rubber. It can be produced by two methods: condensation of 6-hydroxycaproic (6-hydroxyhexanoic) acid and the ring opening polymerisation (ROP) of e-CL [36]. 

**Figure 7:**
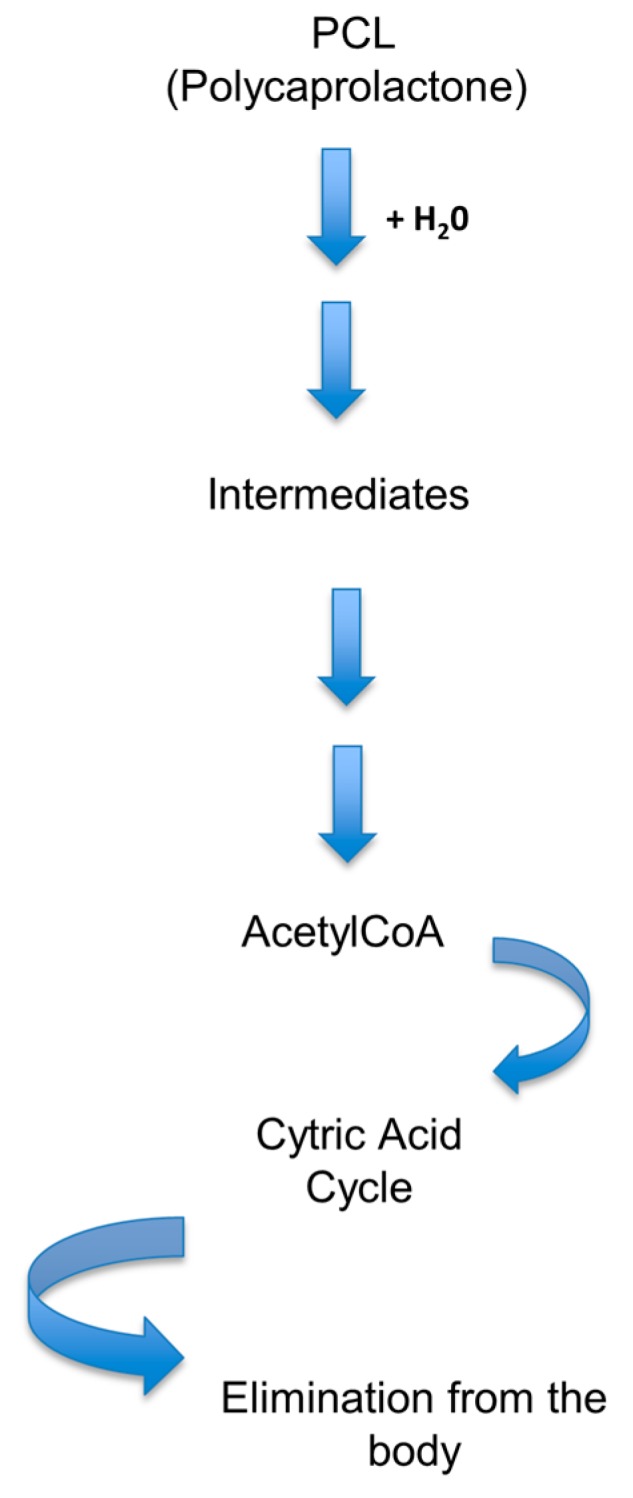
PCL degradation process.

PCL can be degradated in the enviroment by bacteria and fungi, but not in vivo, as the human body lacks the enzymes needed for its biodegradation. The polymer is, however, bioresorbable, even though the process is longer, starting with a hydrolitic degradation (Figure 7) [34]. The degradation time of the homopolymer PCL varies from two to four years depending on the molecular weight, the degree of crystanillity, and the degradation conditions [34,36]. The time of degradation of PCL is longer than those of PLA or PGA, making it more suitable for long-time degradation devices as drug delivery systems with an extended half-life for more than a year period of time [34]. 

The wide blend compatibility, its low melting point, and it solubility properties make this polymer suitable for different biomedical applications, such as tissue engineering [35], wounds dressings [37], or drug delivery systems [34,36].

In a recent study by Berck, R.C.R. et al. (2017), 3D printed tablets loaded with polymeric nanocapsules made of PCL and Eudragit RL100 were produced by using a FDM printer. The extruding temperature was set at 110 °C for Eudragit and 65 °C for PCL. The printing temperature of Eudragit filaments was set at 170 °C and 95 °C for PCL. The settings for the FDM printer were 90 mm/s extruding speed and 100% infill percentage, along with some Eudragit filaments with 50% infill percentage in order to evaluate the influence of filling into the tablets. The drug content (mg/tablet) and the drug loading depended on the type of polymer used: tablets made of Eudragit had a higher drug content and drug loading than those made of PCL, most likely because of their higher swelling indices. Regarding the release profile, Eudragit tablets had a higher release than PCL tablets [38].

## 4. Applications of 3D Printed Drugs

### 4.1. Commercially Available 3D Printed Drugs: Spritam^®^

In 2015, the FDA approved the first 3D printed drug, Spritam^®^ (Aprecia Pharmaceuticals, East Windsor, NJ, USA), containing levetiracetam, an antiepileptic API. The pharmacological efficacy was found to be equivalent with respect to conventional tablets, but with the great improvement that the solubilization time was significantly reduced due to its porous and soluble matrix composition.

Spritam^®^ is marked by Aprecia Pharmaceuticals using the ZipDose technique based on powder bed fusion by the layer-by-layer production system. The first layer consists of the active ingredient and all of the excipients necessary to produce the matrix tablet. Subsequently, a binder liquid is deposited for perfect integration and aggregation between all of the successive and identical layers. The end result is an orodispersible tablet, which dissolves in a few seconds and with a very small amount of water, with a capacity of dosage up to 1000 mg of API [39,40]. This breakthrough highlights the potential of this technology for the development of specialized dosage forms with features not attainable through compression or other conventional manufacturing methods.

### 4.2. Personalized Topical Treatment Devices 

**Figure 8:**
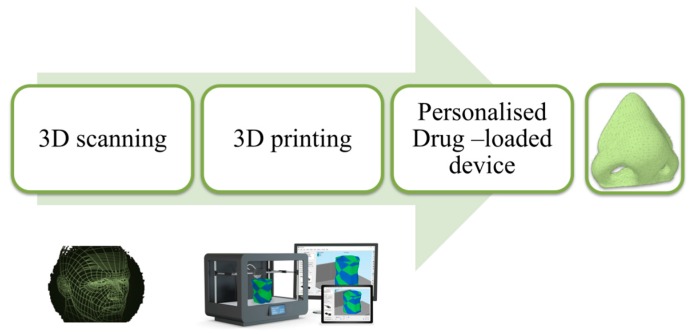
Customized modelling of anti-acne mask [18].

The 3D printing revolution is also feasible in the manufacture of custom, drug-laden devices tailored in shape and size for each patient. Nose-shaped masks, loaded with salicylic acid, intended for anti-acne treatments have been developed in a shortly and efficient manner. The face of the patient was scanned and the captured image was exported to the autocad program, through which the nose section was selected. To produce the three-dimensional model with complete precision, it was necessary to leave the internal part hollow to ensure the perfect adaptation to the patient’s face (Figure 8). After the geometry model was generated, it was printed using two different techniques: FDM and SLA, to determine which one was more favourable in terms of manufacturing, the morphological characteristics of the object, drug release, and the stability during printing.

SLA was the most promising technology for the mask manufacture due to the higher device resolution, allowing for a higher drug loading, and also, for the insignificant degradation of salicylic acid during 3D printing [18]. The highlight of this finding is the wide perspective that exists with the combination of the scanner and 3D printing in the era of treatment personalization based on dosage, size, and shape of specific devices to deal with certain pathologies.

### 4.3. 3D Printing for Cancer Treatment

Traditional chemotherapy has difficulties reaching therapeutic concentrations at the tumor site. This is because most chemotherapeutic drugs have a poor solubility in aqueous media, and therefore, by conventional techniques such as intravenous injection or oral administration, the required concentrations at the tumor site are not achieved. In addition, antineoplastics are usually accumulated in relevant organs such as the liver and heart, causing serious side effects. Thus, local delivery systems would be of great advantage to overcome the deficiencies of conventional chemotherapy.

Currently, the production of patches loaded with 5-fluorouracil, poly (lactic-co-glycolic) acid, and PCL have been successfully printed and implanted directly into a pancreatic cancer. The geometry of the patch and the release kinetics were manipulated, maintaining the drug release for a total of four weeks. After that period, the patch was biodegraded in the body [41]. 

### 4.4. 3D Printed Polypill

**Figure 9:**
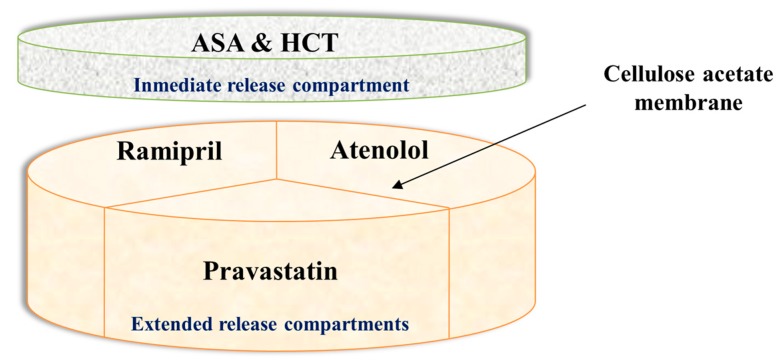
3D printed polypill. Modified from reference [3].

The concept of “polypill” refers to a single tablet that includes the combination of several drugs. Therefore, it provides huge benefits in polymedicated patients, such as the elderly. Different polypills using 3D extrusion printing have been successfully created. As an example, captopril, nifedipine, and glipizide, to treat hypertension and type 2 diabetes, have been manufactured in a single pill by using 3D printing. The technology has moved forward and currently, prototypes including five different types of APIs with different release profiles have been produced [3]. Three APIs (pravastatin, atenolol, and ramipril) were included in the extended release compartment where the drugs were physically separated by a permeable membrane of hydrophobic cellulose acetate. An immediate release compartment containing aspirin and hydrochlorothiazide was deposited on top of the aforementioned compartment (Figure 9).

### 4.5. Applications in Tissue Engineering

Tissue engineering is a multidisciplinary field that is currently focused on two main areas: the development of new methods to repair, regenerate, and replace damaged tissues and organs, and the creation of in vitro tissue models dedicated to the investigation of molecular mechanisms, involved in the progression of the disease to perform drug screening [42].

By conventional techniques, the control of the architecture and the composition of the support along with the shape, size, and distribution of pores is very limited; 3D Bioprinting has become a successful technique to overcome these issues. Using computer aided design, 3D printing enables the construction of tissues from commonly used medical images, such as those obtained by magnetic resonance, X-ray, and computed tomography (CT) scan. 3D Bioprinting prints living cell systems, with or without support by using a specific material called bioink, which would be the equivalent of the extracellular matrix constituents [42]. One of the major issues in bioprinting is to maintain the cell viability both during and after printing. The most suitable techniques with better results are: SLA and printing-based inkjet [43]. An additional problem is the printing of the vascular networks of organs and tissues which still has not been resolved but, it is estimated that in less than 20 years it will be possible to print a complete and perfect heart [23].

At the moment, 3D Bioprinting has been successful in creating knee meniscus, heart valves, artificial ear, as well as designing a custom-made biorreabsorbable trachea which already has been implanted in a neonate with tracheobroncomalacia [23].

## 5. Conclusions

Three-dimensional printing has become a useful and potential tool for the pharmaceutical sector, leading to personalised medicine focused on the patients’ needs. It offers numerous advantages, such as increasing the cost efficiency and the manufacturing speed, since a RP can be done in a matter of minutes. However, there is still a significant barrier to ensure that 3D printed medicines have the same efficacy, safety, and stability as the pharmaceuticals conventionally manufactured by the Pharmaceutical Industry. Regarding the establishment of guidelines, laws, quality systems and safety of use and consumption of 3D printed medicines, it is a great challenge for the regulatory authorities entailing great obstacles, given the traditional requirements by the pharmaceutical sector.

However, the perspective of the regulatory authorities is adapting fast to the real world and patient’s needs. The FDA developed in 2016 a new guidance entitled “Technical Considerations for Additive Manufactured Devices” in order to provide the FDA’s initial thinking on technical considerations associated with AM processes, and recommendations for testing and the characterization for devices that include at least one AM fabrication step [44]. This is just the beginning of the revolution of drug manufacturing techniques.

## Figures and Tables

**Table 1:** Comparison of three-dimensional (3D) Printing Techniques.

3D Printer	CIJ	FDM	PAM	SLA
**Polymer**	Polymer Stabilizer Liquid	Material heat-resistant as melted metals, photo-polymerizable resin and thermoplastic materials	Semi-liquid viscous material	Liquid photopolymer which rapidly solidifies with UV light, as low molecular weight polyacrylate macromers
**Polymer Example**	Tween 20	PVA, PLA, Nylon, ABS, Polyvinyl chloride	Hydroxypropyl methylcellulose (HPMC), Polyacrylate Methocel^®^ E5	Epoxy Resin Acrylic resin PEGDA (liquid photosensitive resin), Propiophenone 2-hydroxy-2-methyl (initiator)
**Drug Type**	Slightly soluble in water and organic solvents	Thermorresistant molecule	Wide variety Non-specific type	Proteins and Peptides
**Drug Example**	Folic Acid	Prednisone, Theophylline, 5-ASA	Nifedipine, Glipizide	BSA (Bovine Serum Albumin)
**Pros**	Works in continuous	Lowest cost, Good mechanical resistance	Manufacture of complex drug delivery systems	Smooth surface due to the use of liquid photopolymers, Manufacture of micro-structures
**Cons**	High energy expenditure and waste generation	Low adequate thermoplastic materials., API degradation due to high temperatures	Use of organic solvents, toxicity and loss of stability	Lack of FDA-approved photosensitive polymers
**Reference**	[4,7,10,11]	[8]	[4]	[4,12]

**Table 2:** Additional information regarding the type of polymers most commonly employed in 3D printing medicines.

Polymer	FDA Approval	Bio-Degradable Polymer	Characteristics	Technique Commonly Employed	Reference
Tween 20 (Polysorbate 20)	✓	✓	GRAS status—Good surfactant properties	CIJ	[10,11,14,15]
Eudragit E100 (Cationic methacrylic ester copolymer)	✓	✓	Soluble under acidic conditions (<pH 5)	Powder Bed Fusion	[4,15,16]
Eudragit RLPO (Copolymer of methacrylic ammonium acid)	✓	✓	Insoluble in permeable water regardless of pH	Powder Bed Fusion	[4,15,16]
MCC (Microcrystalline cellulose)	✓	✓	Used as a disintegrator	FDM	[15,17]
Polyacrylic acid (PAA)	✓	✓	Used as a hydrophilic matrix	FDM	[15,17]
Polyvinyl alcohol (PVA)	✓	✓	Biocompatible water-soluble synthetic polymer capable of swelling upon contact with aqueous fluids.	FDM	[4,12,15,17]
Polyacid-L-lactic (PLLA)	✓	✓	Biodegradable aliphatic polyester that comes from renewable resources such as corn starch, tapioca roots or sugar cane	FDM	[12,15,17,18]
Polyetherimide (PEI)	X	X	Remains unchanged after autoclaving	FDM	[17,19]
Polyphenylsulfone (PPSF)	X	X	Known as RADEL. High heat and chemical resistance.	FDM	[15,19]
Policaprolactone (PCL)	✓	✓	Biocompatible polyester, used in wound dressings, tissue engineering and drug administration	FDM	[15,18,20]
NinjaFlex^®^ (NF)	✓	✓	Thermoplastic polyurethane widely used for regeneration, bone substitution and drug delivery	FDM	[15,20]
PLA flexible variety (FPLA)	✓	✓	Aliphatic polyester with adequate mechanical strength and low toxicity	FDM	[15,18,20]
Methocel^®^ E5 (matrix gel)	✓	✓	Used for immediate release tablets	PAM	[4,15]
Hydroxypropyl Methylcellulose (HPMC)	✓	✓	Used for drug released tablets and polypills	PAM	[4,15,17]
Carbopol^®^ 974P (Polymer crosslinked acrylic acid)	✓	✓	Used for sustained release purposes	PAM	[15,17]
Polyethylene glycol diacrylate (PEGDA)	X	✓	Used as liquid photopolymer	SLA	[4,15,18,20]
Polyethylene glycol (PEG)	✓	✓	Solidifies with the action of a laser beam	SLA	[15,17,20]

**Table 3:** Polyvinyl alcohol properties. Key: Tm (melting point for partially hydrolized PVA); Tm’ (melting point for fully hydrolized PVA); Tg (glass transition temperature); Td (degradation temperature); Viscosity refers to partially hydrolized PVA; Viscosity’ refers to fully hydrolized polymer.

	Tm (˚C)	Tm’ (˚C)	Tg (˚C)	Td (˚C)	Viscosity (mPa·s)	Viscosity’ (mPa·s)	LD_50_ (g/kg)	Drug Loading Examples (% w/w)	Reference
**PVA**	180	220	85	350–450	3.4–52	4.0–60	15–20	0,24; 1,9; 3,9; 8,2	[4,27,28,30]

**Table 4:** Poly(lactic acid) properties. Key: Tm (melting temperature); Tg (glass transition temperature); Td (degradation temperature); Melt viscosity (at 200 **°**C); Melt viscosity’ (at >200 **°**C); Pd (degradation period until 50% or 100% mass loss occur); Deg. Rate (degradation rate of copolymer); Process. T. (processing temperature for the polymer).

	Tm (°C)	Tg (°C)	Td (°C)	Melt Viscosity (Pa·s)	Melt Viscosity’ (Pa·s)	Pd 50% Mass Loss (Months)	Pd 100% Mass Loss (Months)	Deg. Rate of Copolymer (PLA + Polyglicolide) (Months)	Process. T. (°C)	Drug Loading Examples (% w/w)	Reference
PLA	150–175	55	325–500	1000	5100	6–12	>36	5–6	185–190	0,4; 1,9	[7,31,32,33]

**Table 5:** Solubility of PVA, PLA and Poly(caprolactone) (PCL).

	Good Solubility	Low Solubility	Insoluble
**PVA**	Water	Ethanol	Other Organic Solvents
**PLA**	Dioxane, acetonitrile, chloroform, methylene chloride, 1,1,2-trichloroethane and dichloroacetic acid	Ethyl benzene, toluene, acetone and tetrahydrofuran (when cold)	Water, methanol, ethanol, propylene glycol and unsubtituted hydrocarbons
**PCL**	Chloroform, dichloromethane, carbon tetrachloride, benzene, toluene, cyclohexanone and 2-nitropropane	Acetone, 2-butanone, ethyl acetate, dimethylformamide and acetonitrile	Alcohol, petroleum ether and diethyl ether
